# The role thermal physiology plays in species invasion

**DOI:** 10.1093/conphys/cou045

**Published:** 2014-11-10

**Authors:** Amanda L. Kelley

**Affiliations:** Department of Ecology, Evolution and Marine Biology, University of California, Santa Barbara, Santa Barbara, CA 93106-9620, USA

**Keywords:** Critical thermal maximum/upper thermal tolerance limit, ecophysiology, heat shock protein, invasive species, thermal physiology, thermal tolerance

## Abstract

The characterization of physiological phenotypes that may play a part in the establishment of non-native species can broaden our understanding about the ecology of species invasion. Here, an assessment was carried out by comparing the responses of invasive and native species to thermal stress. The goal was to identify physiological patterns that facilitate invasion success and to investigate whether these traits are widespread among invasive ectotherms. Four hypotheses were generated and tested using a review of the literature to determine whether they could be supported across taxonomically diverse invasive organisms. The four hypotheses are as follows: (i) broad geographical temperature tolerances (thermal width) confer a higher upper thermal tolerance threshold for invasive rather than native species; (ii) the upper thermal extreme experienced in nature is more highly correlated with upper thermal tolerance threshold for invasive vs. native animals; (iii) protein chaperone expression—a cellular mechanism that underlies an organism's thermal tolerance threshold—is greater in invasive organisms than in native ones; and (iv) acclimation to higher temperatures can promote a greater range of thermal tolerance for invasive compared with native species. Each hypothesis was supported by a meta-analysis of the invasive/thermal physiology literature, providing further evidence that physiology plays a substantial role in the establishment of invasive ectotherms.

## Abstract (precis)

The work presented here identifies four major physiological traits that have likely played a role in the successful establishment of invasive organisms across taxa.

## Introduction

One of the fundamental questions in invasion ecology is what makes an invading species successful (Elton, 1958). Over 50 years of research addressing this question has illustrated that a variety of life-history traits and other biological characteristics promote invasion success. For example, generalist predatory habits (Snyder and Evans, 2006), dynamic population growth after an initial lag period (Sakai *et al.*, 2001) and superior competitive ability relative to native organisms (Callaway and Ridenour, 2004) are among several of the components that have been shown to facilitate the establishment of non-native species.

To date, much of the research conducted on invasive species has taken the ecosystem- (Gordon, 1998; Dukes and Mooney, 2004) or community-level approach (Kourtev *et al.*, 2002; Sanders *et al.*, 2003), describing the impacts that invaders have on native flora and fauna from a broad-scale perspective. This work has developed and tested hypotheses that have brought about significant advances in our understanding of the unintended consequences of human-mediated invasions. There are, however, many questions that remain unanswered. Despite the extensive literature on the role that physiology has played in the shaping of evolutionary trajectories and biogeographical distributions of native species (Somero, 2002, 2005), there has, until recently, been a dearth of studies that aim to evaluate the physiology of non-native species. Ecophysiology of invasive species is an emerging field that drives to illuminate the physiological mechanisms that play a role in the establishment of non-native species. Narrative reviews of invasion physiology and niche modelling have provided anecdotal evidence of mechanisms that have had broad taxonomic success, but the empirical support for such mechanisms is lacking (Pyšek and Richardson, 2007; Jiménez-Valverde *et al.*, 2011).

In order for invaders to become established in a recipient environment, they must first pass through the ‘ecological filter’ of that environment (Crowl *et al.*, 2008). The ecological filter is composed of two major components, the biotic and the abiotic (Crowl *et al.*, 2008). Invaders new to a recipient habitat must be able to maintain performance and fitness with respect to species interactions such as competition (Levine *et al.*, 2004) and predation (deRivera *et al.*, 2005); failing to do so can prevent establishment or further range expansion. Likewise, for the abiotic component, successful establishment requires invaders to tolerate, through physiological adjustments, abiotic stressors such as temperature, desiccation and disturbance (Olyarnik *et al.*, 2009). Abiotic parameters have been shown to serve as the first ‘ecological filter’ to invasion by non-natives (Olyarnik *et al.*, 2009) and have been proven to prohibit the establishment of invasive populations (Holway *et al.* 2002; Gerhardt and Collinge, 2007). In some cases, abiotic factors can have a greater impact on invasion success than biotic interactions (Dethier and Hacker, 2005).

Temperature is often regarded as the most important abiotic factor in determining species distribution owing to its impact on biochemical and cellular processes (Somero, 2002, 2005; Johnston and Bennett, 2008), which, in turn, affect organismal performance (Pörtner, 2002). Particularly for ectotherms, understanding how an invading organism might respond to variations in environmental temperature can bring about significant insight regarding the patterns and processes of species invasion. Organismal, cellular, genetic and metabolic processes, each play a part in setting the limits of physiological tolerance and are differentially regulated depending on the intensity and amplitude of environmental stress (Prosser, 1991; Johnston and Bennett, 2008). A rich history of research on the thermal physiology of native species provides many measurements that characterize the upper thermal tolerance threshold (UTT) of a species or population, such as LT_50_/CTmax (for review see Lutterschmidt and Hutchison, 1997), and these techniques have recently been applied to the study of invasive species (Kelley *et al.*, 2011; Zerebecki and Sorte, 2011). These measurements allow a comparison of physiological tolerances both within and among species, and can be used to highlight the physiological differences that may exist between native and invasive organisms.

This review provides a framework in which physiology is used to increase our understanding of the mechanisms that influence the successful establishment of non-native organisms. Here, I compare the responses of invasive organisms (those species that have become successfully established in novel environments) vs. native species (those species that have never become established beyond their native habitat ranges). While many organisms (native and non-native alike) use these physiological responses, they are commonly found among invaders and are likely to help such species overcome potential abiotic resistance of recipient habitats. Many of the invasive and native species included in this review do co-occur in areas, while some do not. The goal is to determine empirically what physiological phenotypes invaders have in common, across taxa and habitat types. In doing so, species that have these physiological phenotypes can be identified, and their potential distribution to new habitats monitored by scientists and resource managers alike. I examined four related hypotheses that are not mutually exclusive. The rationale for each hypothesis is explained below.

### Hypothesis 1: thermal width is associated with thermal tolerance

**Figure 1:**
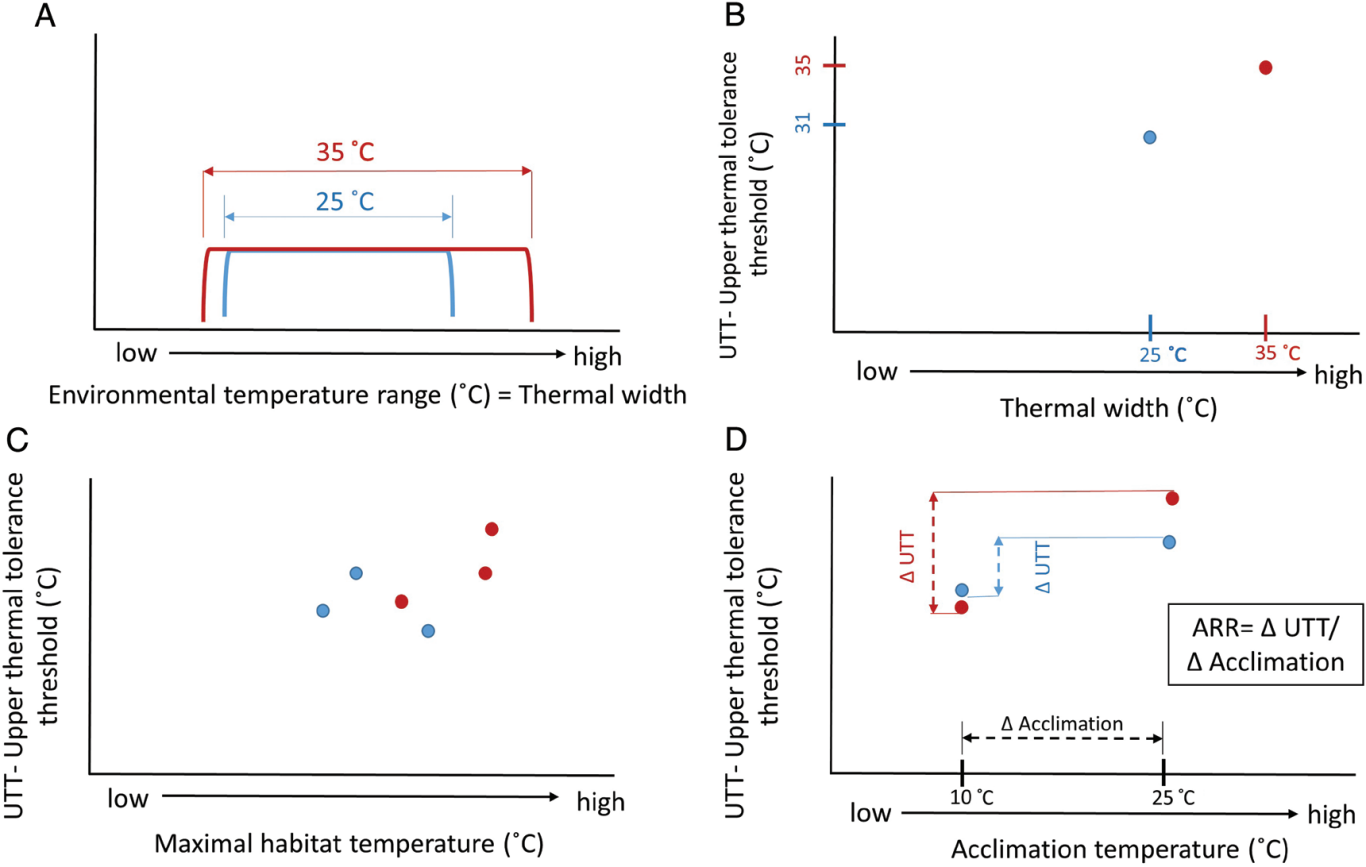
Theoretical diagrams graphically illustrating key concepts. Blue lines/dots represent native species and red lines/dots represent invasive species. (**A**) The thermal width, i.e. the maximum high and minimum low temperature a species experiences in nature, of individual native and invasive species. (**B**) Hypothesis 1; a scatter plot demonstrating the relationship between the thermal width a species experiences (values derived from panel A; invasive thermal width = 35°C, native thermal width = 25°C) and the laboratory-measured upper thermal tolerance threshold (UTT), as described by Zerebecki and Sorte (2011). (**C**) Hypothesis 2; a scatter plot describing the relationship between the maximal habitat temperature a species encounters in nature and the laboratory-measured UTT, as described by Stillman and Somero (2000). (**D**) Hypothesis 3; a graph showing the shift in laboratory-measured UTT after acclimation to a higher temperature, as described by Claussen (1977).

The trait of eurythermality, the ability to maintain physiological function across an extensive range of temperatures, has been theorized as a mechanism of invasive species success (Forcella and Wood, 1984; Rejmánek, 1996; Rejmanek, 2000; McMahon, 2002; Braby and Somero, 2006a, b; Zerebecki and Sorte, 2011). Species that experience a broad ancestral thermal width (Fig. 1A) may be equipped (pre-adapted) for survival, if introduced, in many potential recipient habitats. Often for invading species, this broad tolerance is a product of chronic, oscillating (often daily) thermal perturbation experienced in the native range, which frequently includes high maximal temperatures (Forcella and Wood, 1984; Carveth *et al.*, 2006).

Zerebecki and Sorte (2011) tested the hypothesis that the geographical temperature range (thermal width) that an organism experiences is related to that organism's upper thermal tolerance threshold. Their work compared a small number of invasive and native marine sessile benthic invertebrates in Bodega Harbor, CA, USA, and found that the invasive species, in general, had broader thermal widths as well as higher UTTs when compared with the native species in this study (Zerebecki and Sorte, 2011). Here, I test for concordance of this hypothesis, comparing range widths and UTTs of native and invasive species representing many taxonimic groups, with the *a priori* expectation that invasive species will have a wider thermal width that is more positively correlated with a higher UTT than for native species (Fig. 1B).

### Hypothesis 2: the upper thermal extreme experienced in nature is correlated with the UTT

It has been reported that animals that experience high environmental temperatures have UTTs that are strongly correlated with their respective environments (Stillman and Somero, 2000; Zerebecki and Sorte, 2011). Invasive species often have UTTs that exceed what is physiologically necessary for the environment that they inhabit (Hofmann and Somero, 1996; Braby, 2004; Tomanek and Zuzow, 2010; Zerebecki and Sorte, 2011). This review examines the hypothesis that the relationship between the highest habitat temperature experienced and the UTT is more positively correlated for non-native species than for native ones (Fig. 1C).

### Hypothesis 3: protein chaperone expression is greater in invasive than in native species

Heat shock proteins (HSPs) are molecular chaperones that aid in the refolding of macromolecules that have become denatured due to heat and other stressors (Lindquist, 1986; Feder and Hofmann, 1999; Fink, 1999). Much work evaluating the functional significance of these protein chaperones has led to a clear understanding of their ecological importance (Hochachka and Somero, 2002; Somero, 2002, 2005). The expression of these gene products is thought to be integral to the setting of organismal thermal tolerance limits, and they have been used as molecular indicators to compare the capacity of organisms to tolerate thermal stress (Tomanek and Somero, 1999; Hochachka and Somero, 2002; Somero, 2002, 2005). Induction of HSPs is theorized to be greater for invasive organisms relative to native species (Zerebecki and Sorte, 2011); however, this trend has not yet been identified across taxa. Additionally, it is hypothesized that invaders have a higher HSP induction temperature (*T*_on_) and maximal expression temperature (*T*_max_) than native species (Hofmann and Somero, 1996; Braby, 2004).

### Hypothesis 4: acclimation to warmer temperatures facilitates a greater increase of UTT for invasive species compared with native species

The ability of an organism to make physiological adjustments that alter its thermal tolerance threshold after exposure to different thermal regimes is referred to as acclimation and has been thoroughly explored in the scientific literature (Dietz and Somero, 1992; Buckley *et al.*, 2001; Braby and Somero 2006b; Wada and Matsukura, 2007). It is hypothesized that invaders have the faculty to provide a greater increase to their UTT after acclimation to a warmer temperature than do native species (Fig. 1D). This physiological mechanism may allow an invader to maintain physiological function over a wider range of temperatures relative to a native species (Chown *et al.*, 2007; Slabber *et al.*, 2007; Hofmann and Todgham, 2010).

## Methodological approaches to comparing physiological responses between native and invasive organisms

### Protocol for peer-reviewed literature search

A literature search of peer-reviewed articles was carried out to evaluate the above four hypotheses based on an examination of work that included physiological responses of invasive and native organisms*.* For the literature review, Google Scholar was screened for articles that fit the criteria necessary to test each hypothesis. Search terms that were used are listed as follows: invasive species, non-native species, LT_50_, CTmax, acclimation, upper thermal tolerance, lethal thermal threshold, lethal thermal limit, thermal tolerance, acclimation response ratio, heat shock protein, HSP, protein chaperone, heat stress and thermal stress (for definitions, see Table 1). No Boolean operators were used. For inclusion in this review, only articles that provided the following information were used: laboratory measured LT_50_/CTmax values, laboratory acclimation temperatures, species name, general taxonomy, high and low geographical thermal limits measured *in situ* (in either the native or invasive range) and origin (native or invasive). This search yielded ∼850 results, of which 75 were useful for the analyses (Supplementary Tables S1–S3). For the statistical analyses, both LT_50_ and CTmax values were included and are referred to hereafter as the upper thermal tolerance threshold (UTT) for consistency throughout this article.

**Table 1:** Glossary of terms

**Acclimation**	The physiological response of an organism to environmental change. In an experimental biology context, animals are held at a constant temperature (or other abiotic conditions, e.g. salinity, oxygen content) in the laboratory for a specified amount of time; thereafter, an experimental assay is applied and a specific physiological response measured.
**Acclimation response ratio (ARR)**	A method to compare the response of different populations to thermal acclimation in the laboratory. It is a ratio that takes the difference between the high and low UTT (in degrees Celsius) at each acclimation temperature (ΔUTT, in degrees Celsius) and divides that value by the difference between the high and low acclimation temperatures (Δacclimation, in degrees Celsius). Arithmetically, ARR = ΔUTT (in degrees Celsius)/Δacclimation (in degrees Celsius). For example, an animal with a larger ARR value is able to provide a greater increase their thermal tolerance threshold after acclimation to a higher temperature than an animal that cannot increase or only slightly increases their upper thermal tolerance threshold after acclimation (Fig. 1D).
**Critical thermal maximum (CTmax)**	A laboratory-derived metric of upper thermal tolerance threshold (in degrees Celsius) that uses the onset of muscular spasms/loss of function (resulting in death in an ecological context) as a signal that the thermal threshold has been reached. The arithmetic mean of this value, mean CTmax, is used to determine the average upper thermal tolerance limit of the experimental sample population. This measurement is used to characterize an organism's UTT.
**Ecophysiology**	A discipline that studies the physiological response of organisms to environmental conditions.
**Heat shock proteins (HSPs)**	A suite of protein chaperones that are induced by heat stress. Many of these protein chaperones are involved in the refolding of proteins that have undergone macromolecular degradation due to thermal stress. The expression of heat shock proteins is thought to be a molecular mechanism that underlies an organism's thermal tolerance threshold.
**Invasive, non-native species**	Species that have been introduced outside of their ancestral range through human-mediated dispersal and have successfully maintained self-sustaining populations.
**Lethal temperature (LT**_**50**_**)**	A laboratory-derived metric of upper thermal tolerance threshold (in degrees Celsius) of an experimental sample population that identifies the temperature at which 50% of the animals in the sample population expire. This measurement is used to characterize an organism's UTT.
**Native species**	Species that have never become established beyond their ancestral or native range.
**Recipient range**	The geographical area into which a non-native species has been introduced.
**Thermal width**	The range of temperatures (the maximum high and minimum low) a species has been found to experience in the environment (Fig. 1A).
**Upper thermal tolerance threshold (UTT)**	The discrete temperature at which muscular spasms or death ensue during an acute thermal stress event, which can be measured using the LT_50_ or CTmax assay.

### Hypothesis 1: thermal width associated with thermal tolerance

To test the hypothesis that temperature tolerance increases with thermal width, a regression analysis was used to investigate the relationship between the thermal width (the difference between the upper and lower temperature range experienced by the organism in the environment) and the UTT of native and invasive organisms (as described by Zerebecki and Sorte, 2011; Fig. 1B). Student's unpaired *t*-test was then conducted to compare the thermal width between native and invasive species. Supplementary Table S1 lists the species, general taxonomy, upper and lower environmental thermal limits (in degrees Celsius), thermal range (in degrees Celsius), UTT (in degrees Celsius), origin (native or invasive), acclimation temperature (in degrees Celsius), rate of thermal ramping, life stage and citation(s) for the above data. When multiple temperature ramping rates were used in a study or when different UTT values for one species were found in the literature, the highest UTT value that was recorded was used. For example, *Dreissena polymorpha* and *Dreissena bugensis*, the zebra mussel and quagga mussel, respectively, were tested at several different ramping rates (Mills *et al.*, 1996), but the rate that elicited the highest UTT was used in the analysis.

### Hypothesis 2: the upper thermal extreme experienced in nature is correlated with the UTT

A comparison investigating the relationship between the upper environmental temperature experienced *in situ* and the UTT was carried out (as described by Stillman and Somero, 2000; Zerebecki and Sorte, 2011). A regression analysis using these data was used to assess the relationship between upper lethal thermal tolerance and the highest temperature that each species experienced in nature (Fig. 1C). In order to determine whether UTTs were greater for invasives than natives, Student's unpaired *t*-test was performed. Where applicable, comparisons within taxonomic groups were made between native and non-native species using summary statistics. Supplementary Table S1 lists the upper temperature that each species experiences in nature, as well as the laboratory-derived UTT.

### Hypothesis 3: protein chaperone expression is greater in invasive than in native species

Given the methodological inconsistencies of HSP measurements, e.g. mRNA vs. protein expression, a meta-analysis was not possible. However, there are a small number of research articles that directly compare HSP expression between invasive species and their native competitors. These data were used to test the hypothesis that HSP expression is greater in invasive organisms than in native ones. Also reviewed were the temperature at which induction of the heat shock response occurred (*T*_on_) and the temperature that produced the greatest expression of HSPs (*T*_max_), which have been shown to vary across thermally heterogeneous environments (Tomanek and Somero, 1999). Summary statistics were performed to compare *T*_on_ and *T*_max_ values between native and invasive organisms. Supplementary Table S2 lists the citation, species, origin (native or invasive), type of HSP measured (Hsp70, Hsp90, etc.), gene expression type (e.g. mRNA, protein), maximal HSP expression level measured, *T*_on_, *T*_max_ and the statistical error associated with the measured variable.

### Hypothesis 4: acclimation to warmer temperatures facilitates a greater increase of UTT for invasives compared with native species

To test this hypothesis, the relationship between the range of laboratory temperature acclimation a species experienced and the respective change in UTT was analysed. An appropriate metric for evaluating how acclimation alters upper thermal tolerance is the acclimation response ratio (ARR). The ARR is a ratio that estimates the ability of organisms to modify UTT values at different acclimation temperatures (as described by Claussen, 1977). The ARR was calculated for each species, according to the following equation:

$$\text{ARR}=\frac{\Delta \text{UTT}}{\Delta \text{Acclimation}}$$

where ARR is the acclimation response ratio, ΔUTT is the UTT (in degrees Celsius) after acclimation to a high temperature minus the UTT (in degrees Celsius) after acclimation to a low temperature and Δacclimation is the high acclimation temperature minus the low acclimation temperature (Claussen, 1977; Fig. 1D). For this analysis, only published studies that listed low and high acclimation treatment temperatures and the corresponding laboratory-measured UTT values were included. Mean ARR values were compared between native and invasive species, and Student's unpaired *t*-test was used to test for significant differences between the two groups. Supplementary Table S3 lists the species, general taxonomy, origin (invasive or native), lower and upper acclimation temperatures (in degrees Celsius), the corresponding UTT temperatures after acclimation to two temperatures (in degrees Celsius), Δacclimation (in degrees Celsius), the ΔUTT and the ARR.

### Statistical analysis

All statistical analyses were conducted using Graphpad Prism. Data sets were subjected to Grubb's test for outliers, and those data that were identified as outliers were removed prior to analysis. For the statistical analyses, all data that were not explicitly listed in the text or figure of research articles used in this review were extracted using web plot digitizer (http://arohatgi.info/WebPlotDigitizer/).

## Results

### Hypothesis 1: thermal width is associated with thermal tolerance

**Figure 2:**
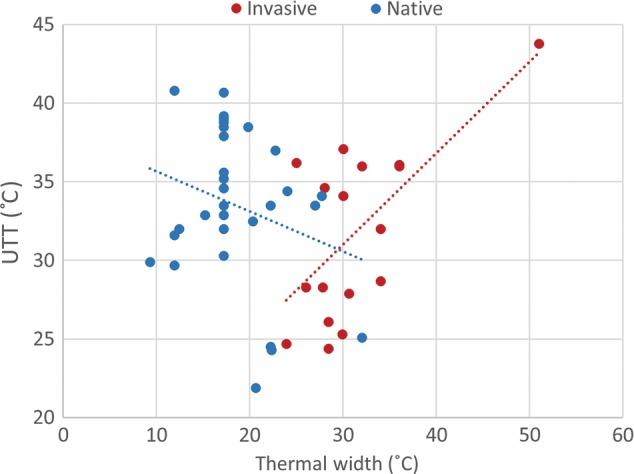
Linear regression illustrating the relationship between geographical thermal width and the UTT of terrestrial and aquatic ectotherms (for invasives, *r*^2^ = 0.43; and for natives, *r*^2^ = 0.07). For references, see Supplementary Table S1.

The thermal width that an invasive population experienced across its geographical range was significantly and positively correlated with its UTT (Fig. 2; *r*^2^ = 0.43, *y* = 0.581*x* + 13.595, *F* = 11.0, *n* = 17, *P* < 0.005). There appeared to be a weak relationship for native organisms (Fig. 2; *r*^2^ = 0.07, *y* = −0.256*x* + 38.226, *F* = 2.0, *n* = 30, *P* = 0.167). Furthermore, the thermal width experienced by native species (mean ± SEM = 19 ± 1.5°C, *n* = 30) was significantly lower than that for non-native ones (mean ± SEM = 31.2 ± 1.5°C; Student's unpaired *t*-test, *t* = 7.7, *n* = 17, *P* < 0.0001).

### Hypothesis 2: the upper thermal extreme experienced in nature is correlated with the UTT

**Figure 3:**
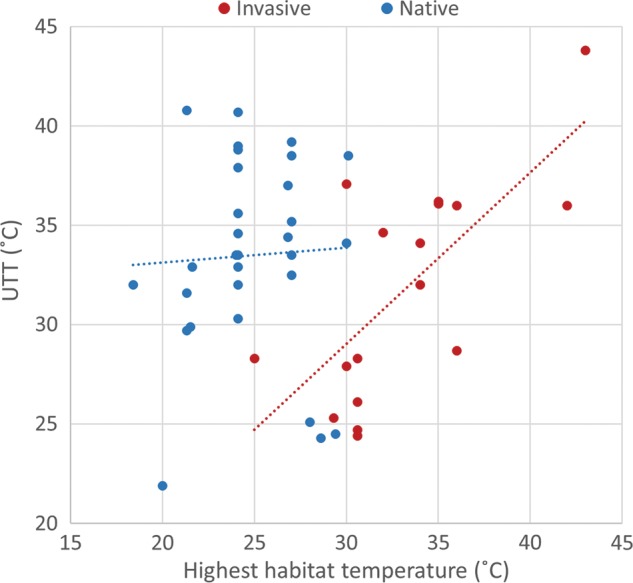
Linear regression illustrating the relationship between highest habitat temperature experienced and the laboratory-measured UTT; for invasive, *r*^2^ = 0.50 and for natives *r*^2^ = 0.002. For references, see Supplementary Table S1.

For invasive ectotherms, a clear trend emerged between the geographical high temperature experienced and the UTT, with *r*^2^ = 0.50 (Fig. 3; *y* = 0.862*x* + 3.147, *F* = 15, *n* = 17, *P* < 0.01). In contrast, there was no detectable pattern between these two variables for native species (Fig. 3; *r*^2^ < 0.01, *y* = 0.075*x* + 31.616, *F* = 0.058, *n* = 30, *P* = 0.81). While the effect of temperature on UTT values differed between the two groups, the range of UTT values was similar for both groups, and there was no significant difference found between the UTT values of natives vs. invasives (Student's unpaired *t*-test, *P* = 0.27). Although the native species used in this analysis had generally high UTT values, further analysis revealed that within taxonomic groups, invaders had slightly higher UTTs than natives of the same taxon. For example, the mean UTT for invasive decapods was 35.1°C, while for native decapods it was 33.5°C. Likewise, for hemiptera, the invasive mean UTT was 43.8°C, while for natives it was 39.6°C.

### Hypothesis 3: protein chaperone expression is greater in invasive than in native species

**Figure 4:**
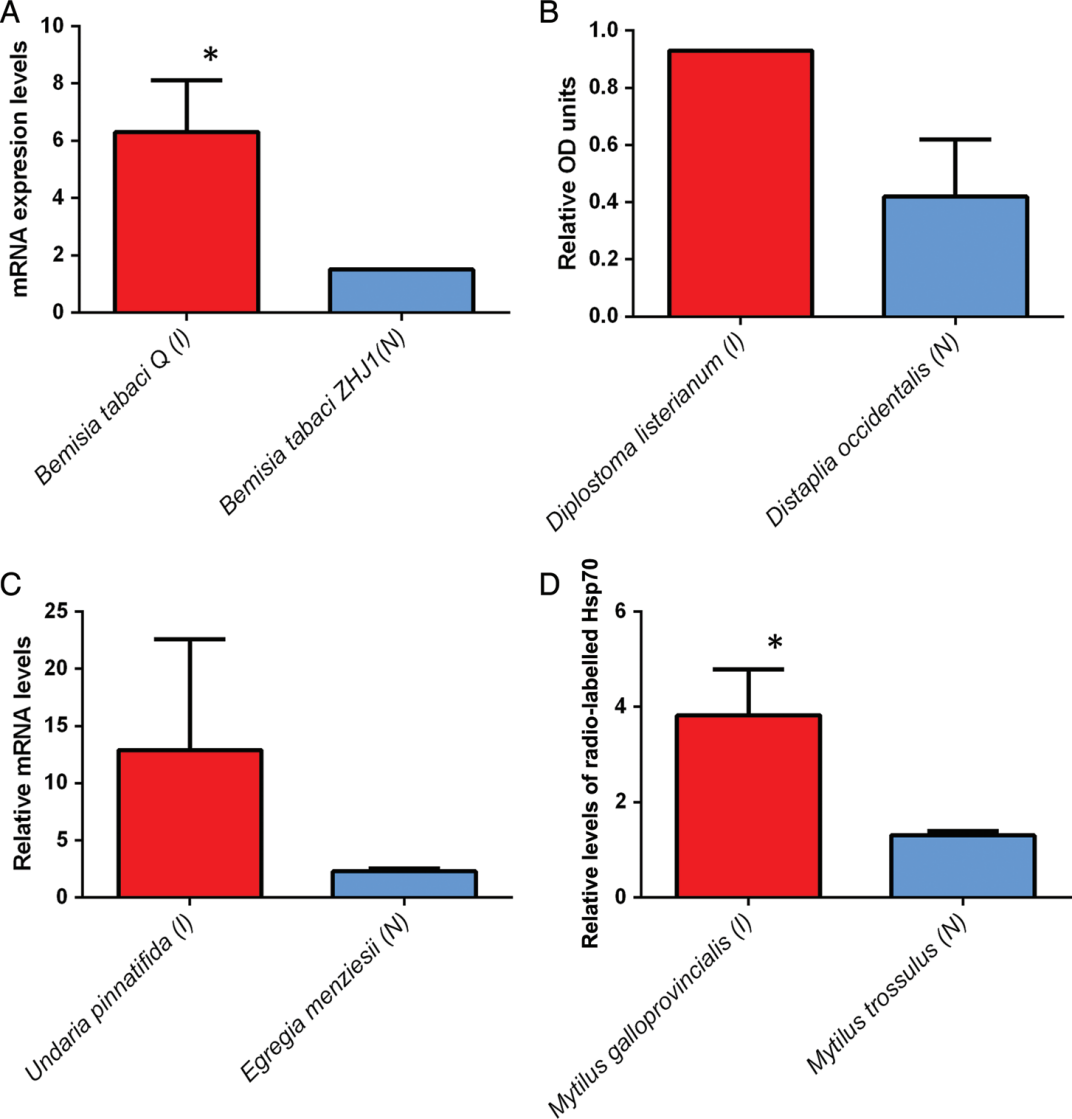
Maximal heat shock protein 70 (Hsp70) expression levels; (I) indicates invasive and (N) indicates native species. (**A**) Hemiptera mRNA levels; error bar represents standard deviation; *P < 0.05. (**B**) Tunicata, relative protein levels; error bar represents standard error. (**C**) Macrophyte, mRNA levels; error bars represent standard error. (**D**) Bivalvia; error bars represent standard error; *P < 0.05. For references, see Supplementary Table S2.

**Figure 5:**
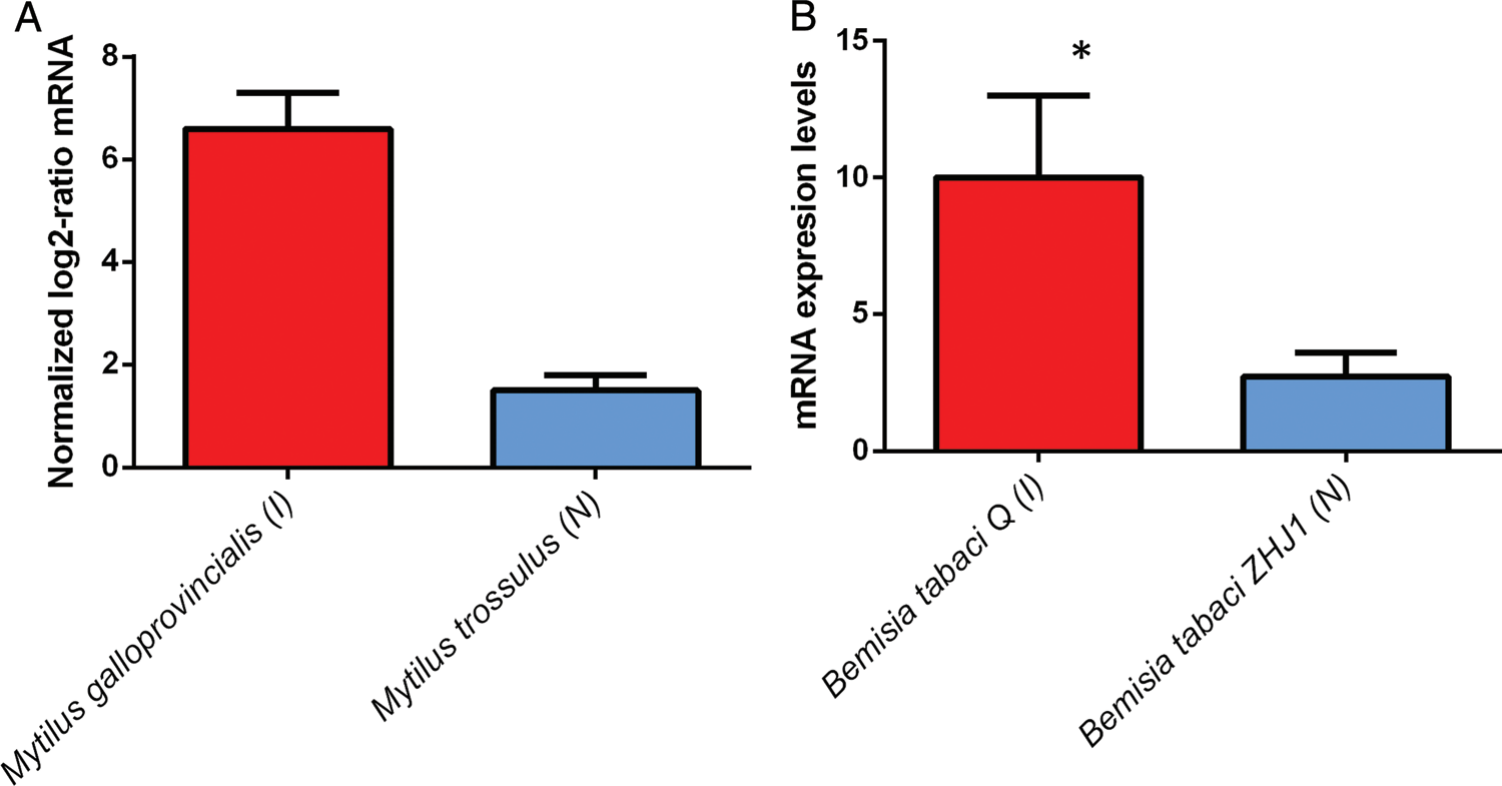
Maximal heat shock protein 20 expression levels; (I) indicates invasive and (N) indicates native species. (**A**) Bivalvia mRNA levels; error bars represent standard error. (**B**) Hemiptera, mRNA levels; error bars represent standard deviation; *P < 0.01. For references, see Supplementary Table S2.

**Figure 6:**
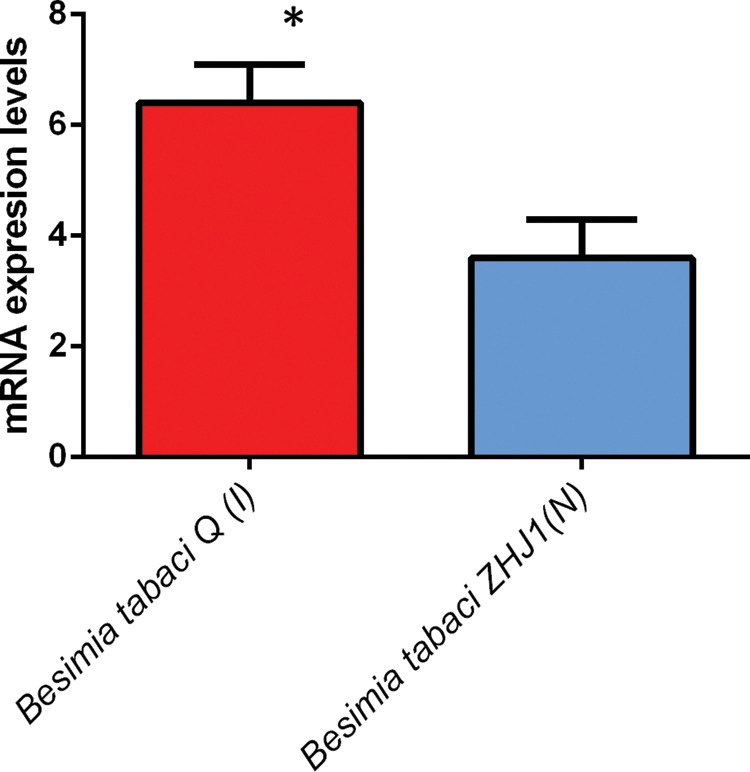
Maximal heat shock protein 90 expression levels for Hemiptera; (I) indicates invasive and (N) indicates native species; error bar represents standard deviation; *P < 0.01. For references, see Supplementary Table S2.

The mean *T*_on_ (the temperature that induced expression for all HSP classes examined) was 29 ± 9.3°C (mean ± SD, *n* = 6) for non-natives, and for native species it was 27.5 ± 10°C (mean ± SD, *n* = 6), yielding a difference of 1.5°C. Likewise, *T*_max_ was 35.3 ± 7.7°C (mean ± SD, *n* = 6) for invasives and 29.5 ± 11.6°C (mean ± SD, *n* = 6) for natives, a difference of 5.8°C. For Hsp70, in two of the four taxa examined, invaders had significantly higher expression levels than did the native species (Fig. 4). The invasive silver leaf white fly (Order hempitera) had significantly higher levels of Hsp20 and Hsp90 than its native congener (Figs 5 and 6).

### Hypothesis 4: acclimation to warmer temperatures facilitates a greater increase of UTT for invasives compared with native species

**Figure 7:**
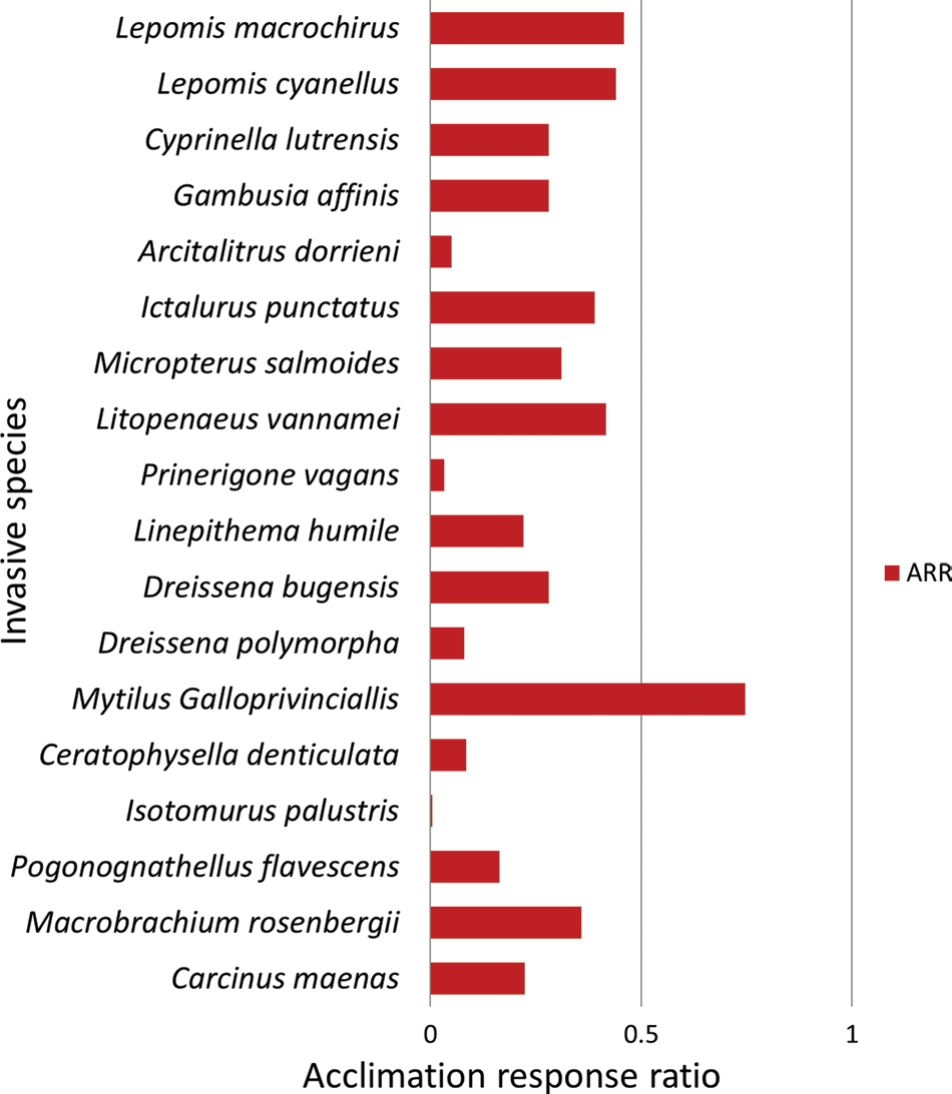
Acclimation response ratio (ARR) of invasive, taxonomically distinct ectotherms. For references, see Supplementary Table S3.

**Figure 8:**
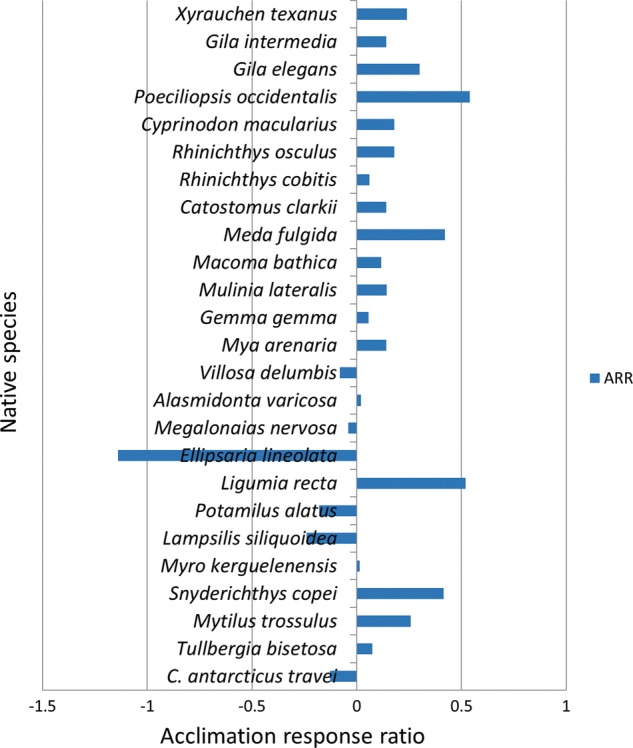
Acclimation response ratio (ARR) of native, taxonomically distinct ectotherms. For references, see Supplementary Table S3.

Invasive organisms responded positively to acclimation, with acclimation to a warmer temperature always resulting in an increase in the UTT, characterized by a positive ARR value (Fig. 7). The ARR for native species was negative in six cases, positive in 19 (Fig. 8). Some native species exhibited negative ARR values, where acclimation to a higher temperature decreased the overall UTT value (Supplementary Table S3).

Student's unpaired *t*-test was used to compare ARR values between native and invasive organisms and showed that they were significantly different (*P* < 0.05, *t* = 2.13). The mean ARR and standard error for invasives and natives was 0.268 ± 0.044 (*n* = 18) and 0.086 ± 0.065 (*n* = 25), respectively. Some native taxonomic groups, e.g. bivalvia, had many negative ARR values (Fig. 8). Conversely, invasive teleosts had ARR values that ranged between 0.28 and 0.46, relatively higher than native teleosts, whose ARR values spanned from 0.06 to 0.44 (Fig. 7; Supplementary Table S3).

## Discussion

The organisms in this study that successfully invaded new regions had several key physiological features that potentially broaden their scope of thermal tolerance. It may be that several of these mechanisms acted synergistically to aid invasives in overcoming abiotic constraints present in the recipient environment. Below is a discussion of how each of these mechanisms may be beneficial to invasive organisms and how they are ecologically relevant in the context of invasion success.

It should be noted that there are several caveats to this approach, and these results should be viewed with caution. First, the rate of heat ramping was not consistent across all UTT experiments, which is reasonable given the different physiologies of the different taxa considered in this review. Second, the acclimation times varied between a week and a month. Variations in either of these parameters can affect the final UTT value, and there are insufficient data to use thermal ramping rate and acclimation temperature as covariates in an analysis. Here, as with the other predictions, the intent is to start examining these mechanisms across taxa to identify the most promising avenues for future experimentation and research.

### Hypothesis 1: thermal width is associated with thermal tolerance

The multispecies comparison revealed that higher temperature tolerance was positively related to the thermal width for invasive but not native species (Fig. 2). The eurythermality of these non-native species, perhaps a byproduct of their fundamental niche, is likely to have played a critical role in their establishment. The fundamental niche of a species can be described as the combination of abiotic variables in which an organism can maintain a population without immigration (Holt *et al.*, 2005). For successful invaders, the abiotic constraints these species were exposed to in the recipient range often bore great similarity to the abiotic parameters experienced in their ancestral range (Holt *et al.*, 2005; Wiens and Graham, 2005). For some invasive species, the niche occupied in the recipient range is nearly identical to that occupied in their home range, as is the case for the invasive decapod *Hemigrapsus sanguineus* (Lohrer *et al.*, 2000). The opposite is true for the invasive green crab, *Carcinus maenas*, as this invasive decapod has invaded variable and spatially disjunct abiotic habitats (Grosholz and Ruiz, 1996). Furthermore, environmental niche modelling has shown that the invasive range of *Carcinus maenas* extends beyond what would be predicted by the native range alone (Compton *et al.*, 2010). Nonetheless, when the ancestral environmental niche is broad, invasive organisms are capable of occupying a wide range of abiotic habitats, which is likely to be a key element in establishing viable, self-sustaining populations in novel habitats.

Other studies examining broad thermal ranges (eurytolerance) and invasion success report results similar to the ones found in this study. For example, Moyle and Marchetti (2006) found that among invasive fishes in California, 66% of successful invasions were found to be fish that were extremely tolerant to high temperatures relative to native species (Moyle and Marchetti, 2006). Also in the genus *Mytilus*, the invasive *Mytilus galloprovincialis* exhibited a thermal niche reflective of its home range (broad thermal range, high UTT), which allowed this species to outcompete the native *Mytilus edulus* at the edge of the *Mytilus edulus* range, facilitating a cryptic invasion along the central Californian coast (Braby, 2004; Braby and Somero, 2006a, b; Lockwood and Somero, 2011).

An invasive organism's ancestral thermal niche, characterized by a discrete thermal range, has been shown to modulate physiological performance on several levels of biological organization (Somero, 2002, 2005). From a molecular standpoint, environmental temperature plays a key role in organismal performance due to its impacts on enzyme kinetics, enzyme–substrate affinity, cell membrane viscosity and the pool of enzymes capable of operating across the thermal range experienced by an organism (Hochachka and Somero, 2002). A loss of function in any of these molecular processes can affect organismal performance, indicating that an invasive organism may be maladapted to the new environment (Hochachka and Somero, 2002).

### Hypothesis 2: the upper thermal extreme experienced in nature is correlated with the UTT

For invasives, an observable trend was identified between the upper thermal temperature experienced *in situ* and the corresponding UTT (*r*^2^ = 0.5; Fig. 3). This study indicates that many invasive species had UTT values that were similar to the upper thermal boundaries present in their ancestral range, allowing them to function closer to their absolute thermal threshold (Stillman and Somero, 2000; Somero, 2005). This is especially true for intertidal ectotherms (Hochachka and Somero, 2002; Jones *et al.*, 2009). The prior habitat characteristics of an invading species can influence the capacity for establishment. For example, intertidal species occupy habitats that vary widely with regard to temperature, salinity, ultraviolet exposure and many other abiotic parameters (Dayton, 1971; Paine and Levin, 1981; Menge and Sutherland, 1987). The occupation of such dynamic environments has led to the evolution of high thermal tolerance thresholds that leave these species poised to pass through the abiotic filter and become established, if introduced to new environments. Many invasive species identified in this review hail from the intertidal range, including *Mytilus galloprovinciallis*, *Carcinus maenas*, *Botryllus schlosseri* and *Didemnum vexillum*.

Although there was no statistical difference between UTTs for native and invasive ectotherms, this is perhaps an artifact of the uneven taxonomic comparison. Many of the native species used in this comparison are terrestrial ectotherms, whereas the majority of invasive species are derived from aquatic habitats (Supplementary Table S2). However, this result makes sense in that the majority of the native species used in the analysis are terrestrial arthropods, a group that has been shown to have high temperature tolerances due to the lack of thermal buffering that is present in aquatic environments (Pörtner, 2002). The results of the present analysis are similar to those of Sorte *et al.* (2013), who found that invasive species were less affected by increases in environmental temperature (as a product of climate change) than native species in aquatic but not terrestrial environments. The results of both studies highlight the difference in upper lethal thermal limits between terrestrial and aquatic ectotherms, with only the aquatic invasive species tending to have a physiological advantage over native ones.

Pairwise comparisons between closely related species found that invasives, in general, had higher UTTs than native species. For example, the invasive *Mytilus galloprovincialis* had an upper critical thermal temperature that was 3 and 4°C higher than the native *Mytilus trossulus* after acclimation to 14 and 21°C, respectively (Braby and Somero, 2006b). This was also the case for springtails from the sub-Antarctic Marion Islands; the invasive springtail *Ceratophysella denticulata* exhibited an UTT of 35.4°C after acclimation to 0.1°C, while the native *Cryptopygus antarcticus* had a lower UTT, 32.7°C, after acclimation to 1.2°C (Slabber *et al.*, 2007).

### Hypothesis 3: protein chaperone expression is greater in invasive than in native species

Owing to the small number of studies available, the statistical power of the pairwise comparisons of protein chaperone expression is low; nevertheless, the expression levels between native and invasive organisms revealed distinct differences in *T*_on_ and *T*_max_ among three classes of heat shock proteins. Below, the functional significance of each HSP is examined, with a discussion of how variation in the expression of these HSPs can affect physiological performance, thereby potentially affecting invasion success.

The inducible isoform of Hsp70 has been widely studied within an ecological context and is thought to be crucial to the establishment and maintenance of the upper thermal tolerance threshold (Sørensen *et al.*, 2003). Indeed, this study showed that *T*_max_ was between 2 and 15°C higher for invasive species than for native species (Supplementary Table S2), indicating that invasives had a greater ability to withstand increased thermal stress. Moreover, non-native species had a higher, *T*_on_ than natives (Supplementary Table S2). For invasive bivalves and hemiptera, *T*_on_ was 2°C higher, while for macrophytes, *T*_on_ was 5°C higher for invasives vs. natives (Supplementary Table S2). These results suggest that the invasive organisms investigated were more tolerant to heat stress in terms of both intensity and duration, as indicated by the higher *T*_on_ and *T*_max_ temperatures. This response may directly support a higher UTT for invasive species, which, in turn, would allow invasives to maintain physiological function at higher environmental temperatures when compared with native species that occupy the same habitat. These results are congruent with work conducted on congeners of the native *Tegula* (marine intertidal snail), where those species that inhabited the upper intertidal zone, and consequently experienced a greater frequency and magnitude of heat stress, had a higher *T*_on_ and *T*_max_ than those species that occupied the mid to low intertidal zones (Tomanek and Somero, 1999). Although the present analysis included only four species, the taxonomic breadth covered was quite broad and captured the response of organisms from four major clades, namely hemiptera, tunicata, bivalvia and macrophyte.

Heat shock protein 20 has been shown to be responsive to both heat and osmotic stresses (Lee *et al.*, 2004; Gracey *et al.*, 2008). The results presented here demonstrate the role that Hsp20 plays as a stress response in two distinct taxa, hemiptera and bivalvia (Fig. 5). For both clades, the invasive species had a greater maximal induction than the native congener (Fig. 5). Functionally, Hsp20 has been shown to inhibit apoptosis (Beere, 2004), and from a mechanistic standpoint, it acts as a chaperone involved in the maintenance of cytoskeletal structure during bouts of environmental stress (Derham and Harding, 1999). In addition, Hsp20 ameliorates the cytotoxic protein aggregation that occurs during heat stress (Horwitz, 1992; Horwitz *et al.*, 1992). For hemiptera, the *T*_on_ of Hsp20 was the same for both native and invasive species, 37°C (Supplementary Table S2). The *T*_max_ for the invasive hemiptera was 41°C, 3°C higher than the native species (Supplementary Table S2). Research by Lockwood *et al.* (2010) found that of all HSPs available, only Hsp20 was upregulated in the warm-adapted, invasive *Mytilus galloprovincialis* when compared with the native *Mytilus trossulus* (Fig. 5; Lockwood *et al.*, 2010). Hence, upregulation of a single HSP class protein may, in part, confer a higher UTT (Braby and Somero, 2006b; Lockwood and Somero, 2011), highlighting the impact that a single HSP class can have in extending the UTT of an organism.

The role of Hsp90 as a molecular chaperone has been well documented (for review, see Pratt, 1998). Heat shock protein 90 overlaps functionally with Hsp70 and is also involved in cell signalling pathways, transcriptional regulation and cell cycle control (Pratt, 1998). Heat shock protein 90 is an important mediator of continued cell cycle progression during bouts of thermal stress (McClellan *et al.*, 2007). In stressful conditions, the ability of an organism to continue cell proliferation (facilitated by the expression of Hsp90) may give an invasive organism a physiological advantage over a native competitor. In hemiptera, Hsp90 expression was significantly greater in the invasive *Bemisia tabaci* biotype Q than in its native congener (Fig. 6). Like the Hsp20 expression in this taxonomic group, Hsp90 had the same *T*_on_, at 35°C, but the *T*_max_ was 2°C higher for *Bemisia tabaci* biotype Q than for the native *Bemisia tabaci* biotype ZHJ1 (Supplementary Table S2).

Taken together, these results indicate that the non-native species had greater expression levels in all HSP classes investigated (Figs 4–6). Furthermore, the studies reviewed showed that in every case, the invaders had a higher UTT, which directly corresponded to higher HSP expression levels in this group.

### Hypothesis 4: acclimation to warmer temperatures facilitates a greater increase of UTT for invasive compared with native species

The ability of ectotherms to make adjustments to organismal thermal tolerance via acclimation has been widely explored and is thought to allow species to persist across highly variable spatiotemporal thermal landscapes (Segal, 1961; Hutchison and Maness, 1979; Roberts *et al.*, 1997; González *et al.*, 2010). The ARRs for invasive species were higher than for the native species examined (0.268 ± 0.044, *n* = 18 and 0.0859 ± 0.0649 *n* = 25, respectively; *P* = 0.04). Results from the present study indicate a divergent response to acclimation. Invasive organisms in all acclimatory conditions increased their UTT after acclimation (Fig. 7). Native species, in contrast, expressed a variety of responses to acclimation; some responded positively, like *Mytilus trossulus*, some responded negatively, as seen in *Ellipsaria lineolata*, and some made no adjustments to their organismal thermal tolerance after acclimation, e.g. *Alasmidonta varicose* (Fig. 8). One possible reason why some native species responded with negative ARR values to higher acclimation (and a resulting lower UTT) is that the higher acclimation temperatures were stressful and very near the UTTs of those species, potentially preventing them from mounting a robust physiological response to acute heat stress (Stillman and Somero, 2000; Stillman, 2003). Moreover, it has been noted that invasive species tend to be more tolerant of high temperatures, and native species are more tolerant of low temperatures when comparing the two across a recipient range (Braby and Somero, 2006b; Slabber *et al.*, 2007). In this scenario, increases in environmental temperature via global climate change may be less problematic or even beneficial for some invasive species, in contrast to native ones (Somero, 2010, 2012).

Acclimatory induced shifts in organismal thermal tolerance are underscored by a broad suite of molecular level processes. These processes include the following: a modification in the transcriptome (Fowler and Thomashow, 2002); a variation in phase behaviour of the lipid bilayer (Hazel, 1995); and a change in the specific isozyme used, to conserve the kinetic activities of *K*_m_ (Michaelis constant) and *K*_cat_ (the number of substrate molecules that can be converted to product) (Hochachka and Somero, 2002; Fields *et al.*, 2006). For the regionally endothermic bluefin tuna, *Thunnus orientalis*, acclimation to two different temperatures prompted a shift in the gene expression, where genes involved in protein biosynthesis, carbohydrate and lipid metabolism were upregulated (Castilho *et al.*, 2009). In *Drosophila melanogaster*, thermal acclimation facilitated reordering of the glycerophospholipids within the lipid bilayer, which helped to maintain membrane function at different temperatures (Overgaard *et al.*, 2008). Fields *et al.* (2006) compared *K*_m_ and *K*_cat_ values of malate dehydrogenase orthologues between the invasive *Mytilus galloprovincialis* and the native *Mytilus trossulus.* This work discovered that the native *Mytilus trossulus* had higher *K*_m_^NADH^ (where NADH is nicotinamide adenine dinucleotide) and *K*_cat_ values of malate dehydrogenase, which pointed to a lower substrate affinity and a higher catalytic rate, reflecting an adaptation to colder temperatures (Fields *et al.*, 2006).

The ability of an invasive organism to acclimate and make physiological adjustments that maintain performance across a range of temperatures is integral to overcoming abiotic resistance within recipient environments. For invasive ectotherms, abiotic constraints pose a substantial barrier that must be surmounted in order for invasive populations to become established (Holway *et al.*, 2002; Gerhardt and Collinge, 2007). For cases when the physiological cost of acclimation inhibits performance, the invader would be at a physiological disadvantage when competing with a native species and would be likely not to establish.

### Conclusions and future directions

In a broader context, distinguishing physiological traits that are common among successful invaders can identify biological characteristics that may predispose a species to become established if introduced (Ricciardi and Rasmussen, 1998; Kolar and Lodge, 2001). In doing so, particular attention can be paid to prevent such species from becoming introduced, or their spread from the zone of introduction can be minimized. For example, the considerable ecological and economic cost that the non-native zebra mussel (*Dreissena polymorpha*; Ludyanskiy *et al.*, 1993) has imposed on the Great Lakes region of the United States has spurred a national interest in the prevention of further expansion of this species. Both federal and local agencies have protocols in place to prohibit the further spread of this prolific invader. The zebra mussel is highly fecund, a trait associated with invasion success (Nalepa and Schloesser, 1993). The Great Lakes may have been less impacted by the zebra mussel if recreational users had been screened for aquatic hitchhikers that exhibited the trait of high fecundity.

Concerning physiological phenotypes that have been correlated with invasion success, an effort can be made by resource managers to identify species that have the physiological capacity to tolerate the abiotic conditions present in the areas they manage. Moreover, if invaders are detected in new habitats, the physiological parameters that these animals exhibit can be used to make predictions concerning their probable distribution within the context of the new environments (Crozier, 2003; Kelley *et al.*, 2013). The over-arching goal of such predictions is to identify potential areas for invasion or range expansion, prevent the establishment non-native species or allow some degree of management of these species once a population has been established. It has been shown that attempts at eradication have a greater degree of success when populations are recently established and relatively small (de Rivera *et al.*, 2006).

The work presented here has identified a methodological approach that compares and elucidates physiological differences between native and invasive species. Nonetheless, it has granted only a correlative relationship between physiological traits that differ between these two groups. Though general trends have been highlighted here, a direct assessment of native and invasive competitors from both aquatic and terrestrial habitats subjected to identical laboratory acclimation, heat ramping rate and upper lethal threshold protocols would further strengthen the support for these hypotheses. This review also establishes the need for further investigations that include more representation from vertebrate and terrestrial species. Additionally, inquiries that look at how invasive organisms might respond to global climate change and what their potential impacts might be on native species, communities and ecosystems need to be undertaken.

## Supplementary material

Supplementary material is available at *Conservation Physiology* online.

## Funding

Financial support was provided by a National Science Foundation Graduate Research Fellowship Grant to A.L.K., grant number 220005, and a National Science Foundation GK-12 Graduate Fellowship to A.L.K., National Science Foundation grant number 0948041.

## Supplementary Material

Supplementary Data
