# Supplementary material for: The role thermal physiology plays in species invasion
Source: Conserv Physiol. 2014 Nov 10;2(1):cou045. doi: 10.1093/conphys/cou045 (PMC4806742; doi:10.1093/conphys/cou045)
Supplement: Supplementary Data [file supp_2_1_cou045__index.html]

The role thermal physiology plays in species invasion — Supplementary Data 

# The role thermal physiology plays in species invasion

## Supplementary Data

Supplementary Data

**Files in this Data Supplement:**

- Supplementary references - docx file
- Supplementary Table 1 - docx file
- Supplementary Table 2 - docx file
- Supplementary Table 3 - docx file
