# Supplementary material for: The role thermal physiology plays in species invasion
Source: Conserv Physiol. 2014 Nov 10;2(1):cou045. doi: 10.1093/conphys/cou045 (PMC4806742; doi:10.1093/conphys/cou045)
Supplement: Supplementary Data [file supp_cou045_cou045supp_references.docx]

**Supplementary References**

Ashton GV, Willis KJ, Burrows MT, Cook EJ (2007) Environmental tolerance of *Caprella mutica*: Implications for its distribution as a marine non-native species. *Mar Envir Res* 64: 305-312.

Ashton KG (2004) Sensitivity of Intraspecific Latitudinal Clines of Body Size for Tetrapods to Sampling, Latitude and Body Size. *Integ Comp Biol* 44: 403-412.

Billman EJ, Wagner EJ, Arndt RE, VanDyke E (2008) Optimal temperatures for growth and upper thermal tolerance of juvenile northern leatherside chub. Western North *Amer Nat* 68: 463-474.

Boos K, Ashton GV, Cook, EJ (2011) The Japanese skeleton shrimp *Caprella mutica*  (Crustacea, Amphipoda): a global invader of coastal waters. In In the Wrong Place-Alien Marine Crustaceans: Distribution, Biology and Impacts (pp. 129-156). Springer Netherlands.

Braby CE, Somero GN (2006b) Following the heart: temperature and salinity effects on heart rate in native and invasive species of blue mussels (genus *Mytilus*). *J Exp Biol* 209: 2554-2566.

Byers J.E, McDowell WG, Dodd SR, Haynie RS, Pintor LM, Wilde SB (2013) Climate and pH Predict the Potential Range of the Invasive Apple Snail (*Pomacea insularum*) in the Southeastern United States. *PLoS ONE* 8:e56812.

Carveth CJ, Widmer AM, Bonar SA (2006) Comparison of upper thermal tolerances of native and nonnative fish species in Arizona. *Trans Amer Fish Soc* 135: 1433-1440.

Claxton WT, Mackie GL (1998) Seasonal and depth variations in gametogenesis and spawning of *Dreissena polymorpha* and *Dreissena bugensis* in eastern Lake Erie. Canadian *J Zool* 76:2010-2019.

Cowling JE, Spicer JI, Weeks JM, Gaston KJ (2003) Environmental tolerances of an invasive terrestrial amphipod, *Arcitalitrus dorrieni* in Britain. *Comp Biochem Phys-Part A: Mole Integ Phys* 136:735-747.

Cox TJ, Rutherford JC (2000) Thermal tolerances of two stream invertebrates exposed to diumally varying temperature. *New Zealand J Mari Freshwater Res***,** 34:203-208.

Cuculescu M, Hyde D, Bowler, K (1998). Thermal tolerance of two species of marine crab, *Cancer pagurus* and *Carcinus maenas*, *J Thermal Biol* 23:107-110.

Currie R, Bennett W, Beitinger T (1998) Critical thermal minima and maxima of three freshwater game-fish species acclimated to constant temperatures. *Envir Biol Fishes* 51:187-200.

Dallas HF, Rivers-Moore NA (2012) Critical Thermal Maxima of aquatic macroinvertebrates: towards identifying bioindicators of thermal alteration. *Hydrobiologia* 679:61-76.

De Wachter B, McMahon BR (1996) Temperature effects on heart performance and regional hemolymph flow in the crab *Cancer magister*.*Comp Biochem Physio Part A: Physiology*, *114*(1), 27-33.

Dı́az Herrera F, Sierra Uribe E, Fernando Bückle Ramirez L, Garrido Mora A (1998) Critical thermal maxima and minima of *Macrobrachium rosenbergii* (Decapoda: Palaemonidae). *J Therm Biol* 23:381-385.

Fowler A, Gerner N, Sewell M (2011) Temperature and salinity tolerances of Stage 1 zoeae predict possible range expansion of an introduced portunid crab, *Charybdis japonica*, in New Zealand. *Biol Invas* 13:691-699.

Henkel S, Hofmann GE (2008) Differing patterns of hsp70 gene expression in invasive and native kelp species: evidence for acclimation-induced variation. *J Appl Phycol* 20:915-924.

Hofmann GE, Somero GN (1996). Interspecific variation in thermal denaturation of proteins in the congeneric mussels *Mytilus trossulus* and *M. galloprovincialis*: evidence from the heat-shock response and protein ubiquitination. *Mari Biol* 126:65-75.

Jumbam K, Terblanche J, Deere J, Somers M, Chown S (2008a) Critical thermal limits and their responses to acclimation in two sub-Antarctic spiders: *Myro kerguelenensis* and *Prinerigone vagans*. *Polar Biol* 31:215-220.

Jumbam K, Jackson S, Terblanche JS, McGeoch MA, Chown S (2008b) Acclimation effects on critical and lethal thermal limits of workers of the Argentine ant, *Linepithema humile*. *J Insect Phys* 54:1008-1014.

Kelley AL, de Rivera CE, Buckley BA (2011) Intraspecific variation in thermal tolerance and morphology of the invasive European green crab, *Carcinus maenas*, on the west coast of North America. *J Exp Mari Biol Ecol* 409:70-78.

Kennedy VS, Mihursky JA (1971) Upper Temperature Tolerances of Some Estuarine Bivalvias. *Chesapeake Sci* 12:193-204.

Kumlu M, Türkmen S, Kumlu M (2010) Thermal tolerance of *Litopenaeus vanname*i (Crustacea: *Penaeidae*) acclimated to four temperatures. *J Therm Biol* 35:305-308.

Lockwood BL, Sanders JG, Somero GN (2010) Transcriptomic responses to heat stress in invasive and native blue mussels (genus *Mytilus*): molecular correlates of invasive success. *J Exp Mari Biol Ecol* 213:3548-3558.

Mills E, Rosenberg G, Spidle A, Ludyanskiy M, Pligin Y, May B (1996) A Review of the Biology and Ecology of the Quagga Mussel (*Dreissena bugensis*), a Second Species of Freshwater Dreissenid Introduced to North America. *Am Zool* 36:271-286.

Muñiz M, Nombela G (2001) Differential variation in development of the B-and Q-biotypes of *Bemisia tabaci* (Homoptera: Aleyrodidae) on sweet pepper at constant temperatures. *Envir Entom* 30(4), 720-727.

Nalepa TF. Schloesser DW (1993) Zebra mussels : biology, impacts, and control. Lewis Publishers, Boca Raton, Fla.

Pandolfo TJ, Cope WG, Arellano C, Bringolf RB, Barnhart MC, Hammer E (2010) Upper thermal tolerances of early life stages of freshwater mussels. *J North Am Benthological Soc* 29:959-969.

Prentice EF, Schneider DE (1979) Respiration and thermal tolerance of the dungeness crab, *Cancer magister* dana. *Comp Biochem Phys Part A: Phys* 63(4), 591-597.

Quinn JM, Steele GL, Hickey CW, Vickers ML (1994) Upper thermal tolerances of twelve New Zealand stream invertebrate species. New Zealand *J Mari Freshwater Res* 28:391-397.

Ramakrishnan V, 2007. Salinity, pH, temperature, desiccation and hypoxia tolerance in the invasive freshwater apple snail, *Pomacea insularum*. University

of Texas at Arlington, Arlington, Texas.

Rawlings T, Hayes K, Cowie R, Collins T (2007) The identity, distribution, and impacts of non-native apple snails in the continental United States. *BMC Evol Biol* 7:97.

Slabber S, Worland MR, Leinaas HP, Chown SL (2007) Acclimation effects on thermal tolerances of springtails from sub-Antarctic Marion Island: Indigenous and invasive species. *J Insect Phys* 53:113-125.

Wang H, Lei Z, Li X, Oetting RD (2011) Rapid cold hardening and expression of heat shock protein genes in the B-biotype *Bemisia tabaci*. *Enviro Entom*, 40(1), 132-139.

Yingying P, Xiaoping DSY (2008) Effects of temperature stress on development, feeding and survival of the apple snail, *Pomacea canaliculata* (Lamarck)[J]. *Acta Phytophylacica Sinica* 3.

Yu H, Wan F, Guo J (2012) Different thermal tolerance and hsp gene expression in invasive and indigenous sibling species of *Bemisia tabaci*. *Biol Invas* 14:1587-1595.

Zaranko DT, Farara DG, Thompson FG (1997) *Canadian J Fish Aquat Sci* 54:809-814.

Zerebecki RA, Sorte CJ (2011) Temperature Tolerance and Stress Proteins as Mechanisms of Invasive Species Success. *PLoS ONE* **6**:e14806.
