# Supplementary material for: The role thermal physiology plays in species invasion
Source: Conserv Physiol. 2014 Nov 10;2(1):cou045. doi: 10.1093/conphys/cou045 (PMC4806742; doi:10.1093/conphys/cou045)
Supplement: Supplementary Data [file supp_cou045_cou045supp_table1.docx]

| **Table 1.** Lists the citation, species, origin- native or invasive, taxon, high and low geographic thermal limits, thermal ramping rate, and UTT value. Those names marked with * represent family level taxonomic identification. | | | | | | | | | | |
| --- | --- | --- | --- | --- | --- | --- | --- | --- | --- | --- |
| **Citations** | **Genus, species** | **Origin** | **Taxon** | **Accl. (˚C)** | | **Rate of ramping** | **Min (˚C)** | **Max (˚C)** | **Thermal Width ˚(C)** | **UTT(˚C)** |
| Zerebecki and Sorte 2011 | *Bugula neritina* | Invasive | Bryozoan | 12 | 4C˚/hr | | 2.2 | 30.6 | 28.4 | 24.4 |
| Zerebecki and Sorte 2011 | *Watersipora subtorquata* | Invasive | Bryozoan | 12 | 4C˚/hr | | 6.7 | 30.6 | 23.9 | 24.7 |
| Zerebecki and Sorte 2011 | *Botrylloides violaceus* | Invasive | Tunicate | 12 | 4C˚/hr | | -0.6 | 29.3 | 29.9 | 25.3 |
| Zerebecki and Sorte 2011 | *Didemnum vexillum* | Invasive | Tunicate | 12 | 4C˚/hr | | 2.2 | 30.6 | 28.4 | 26.1 |
| Zerebecki and Sorte 2011 | *Diplosoma listerianum* | Invasive | Tunicate | 12 | 4C˚/hr | | -0.6 | 30 | 30.6 | 27.9 |
| Zerebecki and Sorte 2011 | *Botryllus schlosseri* | Invasive | Tunicate | 12 | 4C˚/hr | | 2.8 | 30.6 | 27.8 | 28.3 |
| Braby and Somero 2006b | *Mytilus gallaprovincialis* | Invasive | Bivalve | 14 | 6C˚/hr | | 2 | 36 | 34 | 28.7 |
| Fowler et al. 2011 | *Charybdis japonica* | Invasive | Decapod |  |  | | 4 | 34 | 30 | 34.1 |
| Cuculescu et al. 1998, Kelley et al. 2011 | *Carcinus maenas* | Invasive | Decapod | 23 | 4C˚/hr | | -1 | 35 | 36 | 36.1 |
| Yingying 2008 | *Pomacea canaliculata* | Invasive | Gastropoda |  |  | | 6 | 42 | 36 | 36 |
| Nalepa and Schloesser 1993, Mills et al. 1996 | *Dreissena polymorpha* | Invasive | Bivalve | 20 | 4C˚/hr | | 0 | 30 | 30 | 37.08 |
| Mills et al. 1996, Claxton and Mackie 1998 | *Dreissena bugensis* | Invasive | Bivalve | 20 | 4C˚/hr | | 4 | 32 | 28 | 34.63 |
| Ashton 2004, Ashton et al. 2007 | *Caprella mutica* | Invasive | Amphipoda | 14 |  | | -1 | 25 | 26 | 28.3 |
| Ramakrishnan 2007, Rawlings et al. 2007, Byers et al. 2013 | *Pomacea canaliculata* | Invasive | Gastropoda | 30 |  | | 4 | 36 | 32 | 36 |
| Quinn et al. 1994, Zaranko et al. 1997, Cox and Rutherford 2000 | *Potamopyrgus antipodarum* | Invasive | Gastropoda |  |  | | 0 | 34 | 34 | 32 |
| Yu, 2012; Muniz, 2001; Wang, 2011 | *Bemisia tabaci* | Invasive | Hemiptera |  |  | | -8 | 43 | 51 | 43.8 |
| http://www.fisheriesconservationfoundation.org/arcel/admin/uploads/FPLP112.pdf | *Pterois volitans* | Invasive | Teleost |  | 12°C/hr | | 10 | 35 | 25 | 36.2 |
| Zerebecki and Sorte 2011 | *Distaplia occidentalis* | Native | Tunicate | 12 | 4C˚/hr | | -0.6 | 20 | 20.6 | 21.9 |
| Zerebecki and Sorte 2011 | *Ascidia ceratodes* | Native | Tunicate | 12 | 4C˚/hr | | 6.3 | 28.6 | 22.3 | 24.3 |
| Zerebecki and Sorte 2011 | *Bugula neritina* | Native | Bryozoan | 12 | 4C˚/hr | | 7.2 | 29.4 | 22.2 | 24.5 |
| DeWacher, 1996; Prentice, 1979 | *Metacarcinus magister* | Native | Decapod |  |  | | 0 | 27 | 27 | 33.5 |
| Dallas and Rivers-Moore 2012 | *Lymnaeidae** | Native | Gastropoda | 17 | 20°C/hr | | 9.8 | 27 | 17.2 | 39.2 |
| Dallas and Rivers-Moore 2012 | *Paramelitidae** | Native | Amphipoda | 17 | 20°C/hr | | 9.4 | 21.3 | 11.9 | 29.7 |
| Dallas and Rivers-Moore 2012 | *Palaemonidae** | Native | Decapoda | 17 | 20°C/hr | | 9.8 | 27 | 17.2 | 35.2 |
| Dallas and Rivers-Moore 2012 | *Elmidae** | Native | Coleoptera | 17 | 20°C/hr | | 6.9 | 24.1 | 17.2 | 39 |
| Dallas and Rivers-Moore 2012 | *Athericidae** | Native | Diptera | 17 | 20°C/hr | | 6.9 | 24.1 | 17.2 | 38.8 |
| Dallas and Rivers-Moore 2012) | *Simuliidae** | Native | Diptera | 17 | 20°C/hr | | 6.9 | 24.1 | 17.2 | 30.3 |
| Dallas and Rivers-Moore 2012 | *Baetidae** | Native | Ephemeroptera | 17 | 20°C/hr | | 6.9 | 24.1 | 17.2 | 34.6 |
| Dallas and Rivers-Moore 2012 | *Heptageniidae** | Native | Ephemeroptera | 17 | 20°C/hr | | 6.7 | 27 | 20.3 | 32.5 |
| Dallas and Rivers-Moore 2012 | *Leptophlebiidae** | Native | Ephemeroptera | 17 | 20°C/hr | | 6.9 | 24.1 | 17.2 | 33.5 |
| Dallas and Rivers-Moore 2012 | *Telagonodidae** | Native | Ephemeroptera | 17 | 20°C/hr | | 6.4 | 21.6 | 15.2 | 32.9 |
| Dallas and Rivers-Moore 2012 | *Tricorythidae** | Native | Ephemeroptera | 17 | 20°C/hr | | 10.3 | 30.1 | 19.8 | 38.5 |
| Dallas and Rivers-Moore 2012 | *Notonectidae** | Native | Hemiptera | 17 | 20°C/hr | | 6.9 | 24.1 | 17.2 | 40.7 |
| Dallas and Rivers-Moore 2012 | *Pleidae** | Native | Hemiptera | 17 | 20°C/hr | | 9.8 | 27 | 17.2 | 38.5 |
| Dallas and Rivers-Moore 2012 | *Corydalidae** | Native | Megaloptera | 17 | 20°C/hr | | 6.9 | 24.1 | 17.2 | 35.6 |
| Dallas and Rivers-Moore 2012 | *Aeschnidae** | Native | Odonata | 17 | 20°C/hr | | 6.9 | 24.1 | 17.2 | 37.9 |
| Dallas and Rivers-Moore 2012 | *Coenagrionidae** | Native | Odonata | 17 | 20°C/hr | | 9.4 | 21.3 | 11.9 | 40.8 |
| Dallas and Rivers-Moore 2012 | *Notonemouridae** | Native | Plecoptera | 17 | 20°C/hr | | 12.2 | 21.5 | 9.3 | 29.9 |
| Dallas and Rivers-Moore 2012 | *Hydropsychidae** | Native | Trichoptera | 17 | 20°C/hr | | 6.9 | 24.1 | 17.2 | 32.9 |
| Dallas and Rivers-Moore 2012 | *Leptoceridae** | Native | Trichoptera | 17 | 20°C/hr | | 6 | 18.4 | 12.4 | 32 |
| Dallas and Rivers-Moore 2012 | *Petrothrinicidae** | Native | Trichoptera | 17 | 20°C/hr | | 9.4 | 21.3 | 11.9 | 31.6 |
| Dallas and Rivers-Moore 2012 | *Philopotamidae** | Native | Trichoptera | 17 | 20°C/hr | | 6.9 | 24.1 | 17.2 | 32 |
| Kennedy and Mihursky 1971 | *Mya arenaria* | Native | Bivalve | 30 | not listed | | 2.8 | 26.8 | 24 | 34.4 |
| Kennedy and Mihursky 1971 | *Gemma gemma* | Native | Bivalve | 30 | not listed | | 4.1 | 26.8 | 22.7 | 37 |
| Kennedy and Mihursky 1971 | *Mulinia lateralis* | Native | Bivalve | 25 | not listed | | 1.8 | 24 | 22.2 | 33.5 |
| Kennedy and Mihursky 1971) | *Macoma bathica* | Native | Bivalve | 30 | not listed | | 2.3 | 30 | 27.7 | 34.1 |
