# Supplementary material for: The role thermal physiology plays in species invasion
Source: Conserv Physiol. 2014 Nov 10;2(1):cou045. doi: 10.1093/conphys/cou045 (PMC4806742; doi:10.1093/conphys/cou045)
Supplement: Supplementary Data [file supp_cou045_cou045supp_table2.docx]

| **Table 2** Lists the citation, species, origin- native or invasive, Hsp measured, detection type, T_on_ ˚C, maximal induction temperature, T_max_ (˚C), highest expression- fold change/optical densitometry/ normalized expression levels, statistical error ±, type of statistical error. | | | | | | | | | |
| --- | --- | --- | --- | --- | --- | --- | --- | --- | --- |
| **Citation** | **Genus, species** | **Origin** | **Hsp measured** | **Detection type** | **T on (˚C)** | **Tmax (˚C)** | **Highest expression- normalized level** | **Statistical error ±** | **Type of statistical error** |
| Hofmann and Somero 1996 | *Mytilus galloprovincialis* | Invasive | Hsp70 | In vitro radio  labeling | 25 | 28 | 1.31 | .09 | Std. error |
| Zerebecki and Sorte 2011 | *Diplosoma listerianum* | Invasive | Hsp70 | Western blot | 16 | 31 | 0.93 | 0 | Std. error |
| Yu, 2012 | *Bemisia tabaci Q* | Invasive | Hsp70 | mRNA | 39 | 43 | 6.3 | 1.8 | Std. dev. |
| Yu, 2012 | *Bemisia tabaci Q* | Invasive | Hsp90 | mRNA | 35 | 43 | 6.4 | 0.7 | Std. dev. |
| Yu, 2012 | *Bemisia tabaci Q* | Invasive | Hsp20 | mRNA | 37 | 41 | 10 | 3 | Std. dev. |
| Lockwood et al. 2010 | *Mytilus galloprovincialis* | Invasive | Hsp20 | mRNA |  |  | 6.6 | 0.7 | Std. error |
| Henkel and Hofmann 2008 | *Undaria pinnatifida* | Invasive | Hsp70 | mRNA | 22 | 26 | 12.9 | 9.7 | Std. error |
| Hofmann and Somero 1996 | *Mytilus trossulus* | Native | Hsp70 | In vitro radio  labeling | 23 | 25 | 3.83 | .96 | Std. error |
| Zerebecki and Sorte 2011 | *Distaplia occidentalis* | Native | Hsp70 | Western blot | 16 | 16 | 0.42 | 0.2 | Std. error |
| Yu et al. 2012 | *Bemisia tabaci ZHJ1* | Native | Hsp70 | mRNA | 37 | 39 | 1.5 | 0 | Std. dev. |
| Yu et al. 2012 | *Bemisia tabaci ZHJ1* | Native | Hsp90 | mRNA | 35 | 41 | 3.6 | 0.7 | Std. dev. |
| Yu, 2012 | *Bemisia tabaci ZHJ1* | Native | Hsp20 | mRNA | 37 | 39 | 2.7 | 0.9 | Std. dev. |
| Lockwood et al. 2010 | *Mytilus trossulus* | Native | Hsp20 | mRNA |  |  | 1.5 | 0.3 | Std. error |
| Henkel and Hofmann 2008 | *Egregia menziesii* | Native | Hsp70 | mRNA | 17 | 17 | 2.3 | 0.2 | Std. error |
