# Supplementary material for: The role thermal physiology plays in species invasion
Source: Conserv Physiol. 2014 Nov 10;2(1):cou045. doi: 10.1093/conphys/cou045 (PMC4806742; doi:10.1093/conphys/cou045)
Supplement: Supplementary Data [file supp_cou045_cou045supp_table3.docx]

| **Table 3** Lists the citation, species, taxon, origin- invasive or native, lower and upper acclimation temperature (˚C), the corresponding UTT temperatures after acclimation to two temperatures (˚C), Δ acclimation (˚C), Δ UTT, and the ARR- Acclimation response ratio. | | | | | | | | | | |
| --- | --- | --- | --- | --- | --- | --- | --- | --- | --- | --- |
| **Citation** | **Genus, species** | **Taxon** | **Origin** | **Low accl. (˚C)** | **High accl.**  **(˚C)** | **Δ Accl (˚C)** | **Low UTT (˚C)** | **High UTT (˚C)** | **Δ LT50 (˚C)** | **ARR** |
| Kelley et al. 2011 | *Carcinus maenas* | Decapod | Invasive | 6.0 | 23.0 | 17.0 | 31.7 | 35.5 | 3.8 | 0.2 |
| Dı́az Herrera et al. 1998 | *Macrobrachium rosenbergii* | Decapod | Invasive | 20.0 | 32.0 | 12.0 | 37.3 | 41.6 | 4.3 | 0.4 |
| Slabber et al. 2007 | *Pogonognathellus flavescens* | Hexapod | Invasive | -2.2 | 12.7 | 14.9 | 32.7 | 35.1 | 2.4 | 0.2 |
| Slabber et al. 2007 | *Isotomurus palustris* | Hexapod | Invasive | -1.1 | 13.9 | 15.0 | 33.6 | 33.6 | 0.1 | 0.0 |
| Slabber et al. 2007 | *Ceratophysella*  *Denticulata* | Hexapod | Invasive | 0.1 | 15.0 | 15.0 | 35.4 | 36.7 | 1.3 | 0.1 |
| Braby and Somero 2006b | *Mytilus Galloprivinciallis* | Bivalve | Invasive | 14.0 | 21.0 | 7.0 | 25.7 | 30.9 | 5.2 | 0.7 |
| Mills et al. 1996 | *Dreissena polymorpha* | Bivalve | Invasive | 5.0 | 20.0 | 15.0 | 35.0 | 36.2 | 1.2 | 0.1 |
| Mills et al. 1996 | *Dreissena bugensis* | Bivalve | Invasive | 5.0 | 20.0 | 15.0 | 30.9 | 35.1 | 4.2 | 0.3 |
| Jumbam et al. 2008b | *Linepithema humile* | Hymenoptera | Invasive | 15.0 | 25.0 | 10.0 | 37.8 | 40.0 | 2.2 | 0.2 |
| Jumbam et al. 2008a | *Prinerigone vagans* | Arachnida | Invasive | 0.0 | 15.0 | 15.0 | 35.4 | 35.9 | 0.5 | 0.0 |
| Kumlu et al. 2010 | *Litopenaeus vannamei* | Malacostraca | Invasive | 15.0 | 30.0 | 15.0 | 35.9 | 42.2 | 6.3 | 0.4 |
| Currie et al. 1998 | *Micropterus salmoides* | Teleost | Invasive | 20.0 | 30.0 | 10.0 | 35.4 | 38.5 | 3.1 | 0.3 |
| Currie et al. 1998 | *Ictalurus punctatus* | Teleost | Invasive | 20.0 | 30.0 | 10.0 | 36.4 | 40.3 | 3.9 | 0.4 |
| Cowling et al. 2003 | *Arcitalitrus dorrieni* | Amphipod | Invasive | 10.0 | 20.0 | 10.0 | 33.2 | 33.7 | 0.5 | 0.1 |
| Slabber et al. 2007 | *C. antarcticus travei* | Hexapod | Native | 1.2 | 16.2 | 15.0 | 32.7 | 30.8 | -1.9 | -0.1 |
| Slabber et al. 2007 | *Tullbergia*  *bisetosa* | Hexapod | Native | 2.3 | 17.3 | 15.0 | 30.1 | 31.3 | 1.1 | 0.1 |
| Braby and Somero 2006b | *Mytilus trossulus* | Bivalve | Native | 14.0 | 21.0 | 7.0 | 24.2 | 26.0 | 1.8 | 0.3 |
| Braby and Somero 2006b | *Mytilus edulis* | Bivalve | Native | 14.0 | 21.0 | 7.0 | 24.2 | 28.9 | 4.7 | 0.7 |
| Billman et al. 2008 | *Snyderichthys copei* | Teleost | Native | 15.0 | 28.0 | 13.0 | 29.6 | 35.0 | 5.4 | 0.4 |
| Jumbam et al. 2008a | *Myro kerguelenensis* | Arachnida | Native | 0.0 | 15.0 | 15.0 | 35.2 | 35.4 | 0.2 | 0.0 |
| (Pandolfo et al. 2010) | *Lampsilis siliquoidea* | Bivalve | Native | 22.0 | 27.0 | 5.0 | 35.6 | 34.4 | -1.2 | -0.2 |
| Pandolfo et al. 2010 | *Potamilus alatus* | Bivalve | Native | 22.0 | 27.0 | 5.0 | 35.0 | 34.1 | -0.9 | -0.2 |
| (Pandolfo et al. 2010) | *Ligumia recta* | Bivalve | Native | 22.0 | 27.0 | 5.0 | 32.5 | 35.1 | 2.6 | 0.5 |
| Pandolfo et al. 2010 | *Ellipsaria lineolata* | Bivalve | Native | 22.0 | 27.0 | 5.0 | 38.8 | 33.1 | -5.7 | -1.1 |
| Pandolfo et al. 2010 | *Megalonaias nervosa* | Bivalve | Native | 22.0 | 27.0 | 5.0 | 34.2 | 34.0 | -0.2 | 0.0 |
| Pandolfo et al. 2010 | *Alasmidonta varicosa* | Bivalve | Native | 22.0 | 27.0 | 5.0 | 35.0 | 35.1 | 0.1 | 0.0 |
| Pandolfo et al. 2010 | *Villosa delumbis* | Bivalve | Native | 22.0 | 27.0 | 5.0 | 34.6 | 34.2 | -0.4 | -0.1 |
| Kennedy and Mihursky 1971 | *Mya arenaria* | Bivalve | Native | 5.0 | 30.0 | 25.0 | 30.9 | 34.4 | 3.5 | 0.1 |
| Kennedy and Mihursky 1971 | *Gemma gemma* | Bivalve | Native | 5.0 | 30.0 | 25.0 | 35.6 | 37.0 | 1.4 | 0.1 |
| Kennedy and Mihursky 1971 | *Mulinia lateralis* | Bivalve | Native | 2.0 | 25.0 | 23.0 | 30.2 | 33.5 | 3.3 | 0.1 |
| Kennedy and Mihursky 1971 | *Macoma bathica* | Bivalve | Native | 5.0 | 30.0 | 25.0 | 31.2 | 34.1 | 2.9 | 0.1 |
| Kennedy and Mihursky 1971 | *Gambusia affinis* | Teleost | Invasive | 25.0 | 30.0 | 5.0 | 40.7 | 42.1 | 1.4 | 0.3 |
| Kennedy and Mihursky 1971 | *Cyprinella lutrensis* | Teleost | Invasive | 25.0 | 30.0 | 5.0 | 39.5 | 40.9 | 1.4 | 0.3 |
| Carveth et al. 2006 | *Agosia chrysogaster* | Teleost | Native | 25.0 | 30.0 | 5.0 | 38.9 | 41.1 | 2.2 | 0.4 |
| Carveth et al. 2006 | *Lepomis cyanellus* | Teleost | Invasive | 25.0 | 30.0 | 5.0 | 39.3 | 41.5 | 2.2 | 0.4 |
| Carveth et al. 2006 | *Lepomis Macrochirus* | Teleost | Invasive | 25.0 | 30.0 | 5.0 | 37.3 | 39.6 | 2.3 | 0.5 |
| Carveth et al. 2006 | *Meda fulgida* | Teleost | Native | 25.0 | 30.0 | 5.0 | 37.0 | 39.1 | 2.1 | 0.4 |
| Carveth et al. 2006 | *Catostomus clarkia* | Teleost | Native | 25.0 | 30.0 | 5.0 | 36.9 | 37.6 | 0.7 | 0.1 |
| Carveth et al. 2006 | *Rhinichthys cobitis* | Teleost | Native | 25.0 | 30.0 | 5.0 | 36.5 | 36.8 | 0.3 | 0.1 |
| Carveth et al. 2006 | *Rhinichthys*  *osculus* | Teleost | Native | 25.0 | 30.0 | 5.0 | 36.0 | 36.9 | 0.9 | 0.2 |
| Carveth et al. 2006 | *Cyprinodon macularius* | Teleost | Native | 25.0 | 30.0 | 5.0 | 41.8 | 42.7 | 0.9 | 0.2 |
| Carveth et al. 2006 | *Poeciliopsis occidentalis* | Teleost | Native | 25.0 | 30.0 | 5.0 | 39.4 | 42.1 | 2.7 | 0.5 |
| Carveth et al. 2006 | *Gila elegans* | Teleost | Native | 25.0 | 30.0 | 5.0 | 38.7 | 40.2 | 1.5 | 0.3 |
| Carveth et al. 2006 | *Gila intermedia* | Teleost | Native | 25.0 | 30.0 | 5.0 | 38.3 | 39.0 | 0.7 | 0.1 |
| Carveth et al. 2006 | *Xyrauchen texanus* | Teleost | Native | 25.0 | 30.0 | 5.0 | 39.1 | 40.3 | 1.2 | 0.2 |
